# The MUC5B Mucin Is Involved in Paraquat-Induced Lung Inflammation

**DOI:** 10.1155/2020/7028947

**Published:** 2020-07-16

**Authors:** Hao Sun, Yunfei Jiang, Yang Song, Xiaomin Zhang, Jun Wang, Jinsong Zhang, Jian Kang

**Affiliations:** ^1^Department of Emergency, Jiangsu Province Hospital, the First Affiliated Hospital of Nanjing Medical University, Nanjing, China; ^2^Department of Emergency, Nanjing Drum Tower Hospital, the Affiliated Hospital of Nanjing University Medical School, Nanjing, China; ^3^Key Lab of Modern Toxicology, Ministry of Education, Department of Toxicology, School of Public Health, Nanjing Medical University, Nanjing, China

## Abstract

**Objective:**

Paraquat (PQ), a widely used toxic herbicide, induces lung inflammation through mechanisms that remain incompletely understood. In a previous study, we found that the plasma MUC5B mucin level was implicated in PQ poisoning in patients. Here, we hypothesize that MUC5B is a critical mediator in PQ-induced cell inflammation.

**Methods:**

A mouse model of PQ-induced lung injury was used to examine the MUC5B expression level. A549 cells (alveolar epithelial cells line) were exposed to PQ in dose-dependent and time-dependent manners. Cell viability was detected by CCK-8 assays. The expression levels of MUC5B were examined by dot blot enzyme-linked immunosorbent assay (ELISA) and RT-qPCR. Western blotting was used to detect the levels of proteins in the MAPK and NF-*κ*B pathways. Inflammatory factors in the cell culture medium were measured by ELISA. NF-*κ*B and MAPK pathway inhibitors and MUC5B siRNA (siMUC5B) were used to determine the function of MUC5B. Finally, N-acetyl-cysteine (NAC) was added and its regulatory effect on the MAPK-NF-*κ*B-MUC5B pathway was examined in PQ-induced cell inflammation.

**Results:**

MUC5B was significantly upregulated accompanying the increases in TNF-*α* and IL-6 secretion following PQ treatment in mouse and also in A549 cells after treatment with 50 *μ*M PQ at 24 hours. Furthermore, MAPK and NF-*κ*B pathway inhibitors could dramatically decrease the expression of MUC5B and the secretion of TNF-*α* and IL-6. Importantly, siMUC5B could significantly attenuate the secretion of TNF-*α* and IL-6 induced by PQ. As expected, the addition of NAC efficiently suppresses the TNF-*α* and IL-6 secretion stimulated from PQ and also downregulated ERK, JNK, and p65 phosphorylation (ERK/JNK MAPK and NF-*κ*B pathways) as well as MUC5B expression.

**Conclusion:**

Our findings suggest that MUC5B participates in the process of PQ-induced cell inflammation and is downstream of the NF-*κ*B and MAPK pathways. NAC can attenuate PQ-induced cell inflammation at least in part by suppressing the MAPK-NF-*κ*B-MUC5B pathway. These results nominate MUC5B as a new biomarker and therapeutic target for PQ-induced lung inflammation.

## 1. Introduction

Paraquat (PQ, 1, 1′-dimethyl-4, 4′-bipyridinium) is a highly toxic herbicide that is used worldwide, especially in developing countries [1, 2]. In the last several years, the incidence of PQ poisoning by accident or suicide has increased in Asia, notably in China [3]. Recent studies have reported that PQ cytotoxicity involves reactive oxygen species (ROS) generation, inflammation. and improper epithelial to mesenchymal transition (EMT) [4–6], although the underlying mechanisms remain poorly characterized.

Mucins (MUCs) are a group of highly glycosylated proteins that are classified into membrane-associated MUC and secretory MUC according to their characteristics [7, 8]. MUCs, also innate immune barriers in the lung, play a protective role in normal airway defense. MUC5B, a type of secretory mucin, is the most important component of the respiratory secretory system. It is secreted by the goblet cells and mucous gland cells of the submucosal glands in the human airway and is an important risk factor for idiopathic pulmonary fibrosis [9–11]. In recent years, various studies have shown that the expression of MUC5B is related to the occurrence of lung injury [12, 13]. Our previous study found that the expression of MUC5B was related to PQ poisoning [14]. In PQ poisoning patients, the concentration of MUC5B in the plasma was increased and correlated to the prognosis of the patients, and these results were consistent with findings from a study of idiopathic pulmonary fibrosis (IPF) patients [15]. Therefore, the regulation and functional role of MUC5B in PQ poisoning deserves further study.

N-Acetyl-cysteine (NAC), a commonly used clinical expectorant, is a precursor that supplies bioavailable cysteine for glutathione replenishment and has mucolytic and antioxidant effects; NAC also leads to the generation of glutathione (GSH) in the body [16–18] and promotes a number of functions in the lung repair process, such as enhancing cell proliferation, promoting migration, and wound healing [19]. Both the Chinese Expert Consensus [20] and the Korean Guidelines [21] recommended that PQ poisoning patients could use NAC as a treatment since the mucolytic and antioxidant effects of NAC have been demonstrated profoundly [22, 23]; however, its anti-inflammatory properties still need to be proven effective, and the underlying mechanism has not been fully elucidated.

The purpose of this study was to examine whether MUC5B participates in PQ-induced cell inflammation and whether NAC can inhibit PQ-induced inflammation by regulating MUC5B expression besides its mucolytic effect on MUC5B. Our results establish a critical function of MUC5B in PQ-induced inflammation and its reversal by NAC, suggesting that MUC5B could serve as a biomarker and therapeutic target for PQ-induced lung toxicity.

## 2. Methods and Materials

### 2.1. Paraquat-Induced Lung Injury Animal Model

Referring to our previous study [24], male C57BL/6J mice (8–9 wk. old) were administered PQ (Sigma-Aldrich, MO, USA) at a single dose of 0.02 mg per mouse (PQ diluted in 50 *μ*l of sterile saline buffer) via intratracheal aerosolization (Model IAIC microsprayer, High Pressure Syringe Model FMJ-250, Penn-Century, PA, USA). Control animals received an equal volume of sterile saline. Mice were sacrificed 3 days or 7 days post PQ administration. Lung injury was assessed by measuring the static compliance, cell count, and neutrophil percentage of bronchoalveolar lavage fluid (BALF) and lung histopathological changes from hematoxylin and eosin (HE) staining. Animal study was approved by the Institutional Animal Care and Use Committee of Nanjing Medical University (NMU; Jiangsu, China) (permit number: IACUC-1712010).

### 2.2. Immunohistochemistry for Muc5b

Immunohistochemistry (IHC) for the detection of Muc5b in formalin-fixed paraffin-embedded mouse lung tissue was performed with an Elivision plus Polymer HRP IHC Kit (Cat. #: 9901; MXB, Nanjing, China) according to the manufacturer's instructions. The sections were incubated with a mouse Mucin-5b antibody (Cat. #: sc-135508, Santa Cruz Biotechnology, CA, USA). The stained sections were viewed under the microscope (Nikon Eclipse C1, Tokyo, Japan) at 100–400x magnification. The slides were scanned by a MIRAX Desk Digital Slide Scanner (Zeiss, Gottingen, Germany).

### 2.3. Cell Culture and siRNA Transfection

A549 human lung adenocarcinoma epithelial cells were obtained from the Cell Bank of the Chinese Academy of Sciences. Cells were cultured in 1640 medium (Gibco, Suzhou, China) containing 10% fetal bovine serum (Sciencell, San Diego, California, USA) and 1% antibiotics (100 U/ml penicillin, 0.1 mg/ml streptomycin) in a humidified incubator at 37°C with 5% CO_2_. siRNA against MUC5B (siMUC5B) and control siRNA (synthesized by GenePharma Company, Shanghai, China) were transfected with the riboFECT™ CP Transfection Kit (RiboBio, Guangzhou, China) according to the manufacturer's instructions.

### 2.4. Cell Model, Application of Inhibitors, and NAC Treatment

To study the effect of PQ on A549 cells, PQ was diluted with a phosphate-buffered saline solution (50000 *μ*M). To obtain various final concentrations of working solution (10, 25, 50, 100, 200, and 400 *μ*M) for the dose-dependent experiment, diluted PQ was further diluted with culture medium and added to A549 cells for 24 h. For the time-dependent experiment, cells were incubated with 50 *μ*M PQ and collected at 1, 3, 6, 12, and 24 h for the following experiments. For pathway evaluation, cells were treated with 50 *μ*M PQ for shorter times (0, 5, 15, 30, 45, and 60 min). The ERK/JNK MAPK-NF-*κ*B pathway inhibitors SP600125 (SP), SCH772984 (SCH), SB203580 (SB) and BAY 11-7082 (BAY), and NAC were added to the cells in advance for 2 h and then replaced with culture medium. PQ and NAC were purchased from Sigma-Aldrich Company (Sigma, St. Louis, MO, USA). The inhibitors SP, SCH, SB, and BAY were obtained from Selleck (Selleck, Shanghai, China).

### 2.5. Cell Viability

CCK-8 assays were utilized to determine the survival rate of A549 cells. Cells were cultured in 96-well plates at a concentration of 1 × 10^4^ cells per well. After treatment, 10 ml (10% of the cell culture medium) of the CCK-8 reagent (Dojindo, Kumamoto, Kyushu, Japan) was added to each well to react for 2 h. Finally, the absorbance (A) was measured at 450 nm using a microplate reader. There were 6 parallel experiments for every group.

### 2.6. 96-Well Dot Blot Enzyme-Linked Immunosorbent Assay (ELISA) Analysis

A dot blot ELISA analysis of the protein samples was performed using an S&S MINIFOLD I dot blot filtration manifold. Samples were lysed with RIPA buffer. After centrifugation (12000 rpm, 4°C, 10 min), the protein concentrations were measured by BCA protein assay kits with BSA as the standard, and the samples were diluted to 0.04 *μ*g/*μ*l. The diluted samples (100 *μ*l) were added to the wells and ran in duplicate. The protein samples were transferred onto PVDF membranes, which were then blocked with 5% milk in PBST for one hour at room temperature and incubated at 4°C with a MUC5B primary antibody (Santa Cruz, CA, USA) diluted 1 : 5000 with 2.5% milk overnight. After washing, secondary goat anti-rabbit IgG biotin conjugate in 2.5% milk (1 : 2000 dilution) was applied for one hour at room temperature, followed by incubation with strep-HRP (Life Technologies, Carlsbad, CA, USA) after washing. The blots were developed using a horseradish fluoro-illuminescence detection protocol and SuperSignal Dura Extended Duration Substrate (Pierce, Rockford, IL). For each wash, the membranes were incubated with PBST for 3 × 15 min at room temperature.

### 2.7. Western Blotting Analysis

In vivo study, the upper part of the left lung lobe was homogenized in 1% NP40 lysis buffer (Beyotime, Shanghai, China) using an Atpio homogenizer (Xianou-24, Nanjing, China). After centrifugation (4°C, 12000 rpm, for 15 min), the supernatants were measured using a Pierce™ BCA Protein Assay Kit (Thermo Fisher Scientific, Rockford, IL, USA). For in vitro study, samples from the cells were lysed with RIPA buffer. After centrifugation (12000 rpm, 4°C, 10 min), the protein concentrations were aspirated for the BCA assay. Protein samples (30 *μ*g) were separated by 10% SDS-PAGE and transferred onto polyvinylidene fluoride (PVDF) membranes (Millipore Corporation, Billerica, MA, USA). After washing, the membranes were blocked with 3% bovine serum albumin (BSA) in TBST and incubated for 1 h at room temperature. Then, the membranes were incubated with primary antibodies (Supplementary Table 1) at 4°C overnight. After washing, the membranes were incubated with an appropriate HRP-conjugated secondary antibody for 2 h. For each wash, the membranes were incubated with TBST for 5 × 5 min at room temperature. Quantification were performed using the ChemiDoc XRS+ system (Bio-Rad, Berkeley, CA, USA) after adding an enhanced chemiluminescence reagent (EMD Millipore; catalog number WBKLS0500) to the surfaces of the membranes.

### 2.8. Real-Time Quantitative PCR

The mRNA expression of MUC5B, or the cytokines TNF-*α* and IL-6, was analyzed by quantitative real-time quantitative PCR (qPCR), and GAPDH was used as an internal control. Total RNA was extracted from A549 cells or from lung tissues using RNAiso Plus (Takara Biotechnology, Co. Ltd. Dalian, China), and reverse transcription was carried out with the PrimeScriptTM RT Master Mix Kit (Takara, Dalian, China) according to the manufacturer's protocol. RNA quantification and quality check were performed with NanoDrop and Agilent 2100 Bioanalyzer (Agilent Technologies, Santa Clara, CA, USA) according to the manufacturer's instructions. Total RNA using qPCR was performed using a SYBR Green kit (Takara Biotechnology, Co. Ltd., Dalian, China). The PCR conditions were as follows: 95°C for 90 s, followed by 40 cycles of 95°C for 5 s, 55°C for 30 s, and 72°C for 60 s. The following primers (Table 1) were used and purchased from TsingKe Biological Technology (Nanjing, China):

### 2.9. ELISA

The levels of the cytokines TNF-*α* and IL-6 in both BALF and serum were measured using mouse ELISA kits (Quantikine ELISA TNF-*α* (MTA00B) and IL-6 (M6000B) Immunoassay, R&D Systems, Minneapolis, MN, USA) while extracellular levels of TNF-*α* and IL-6 from A549 cells were assessed by ELISA kits (Quantikine ELISA TNF-*α* (DTA00D) and IL-6 (D6050) Immunoassay for Human, R&D Systems, Minneapolis, MN, USA) according to the manufacturer's instructions. After exposure to 50 *μ*M PQ for 24 h, the culture medium was collected, and TNF-*α* and IL-6 levels were determined after cell debris was removed by centrifugation.

### 2.10. Statistical Analysis

All data are expressed as the means ± S.D. All analyses were performed with GraphPad Prism 6. Comparisons between different groups were conducted using 2-tailed Student's *t*-tests. ANOVA was used for the analysis of multiple comparisons. Values of probability (*p*) less than 0.05 were considered to be significantly different.

## 3. Results

### 3.1. PQ Activated the NF-*κ*B Pathway and Induced Muc5b Expression, TNF-*α*, and IL-6 Production in Mice

**Figure 1:**
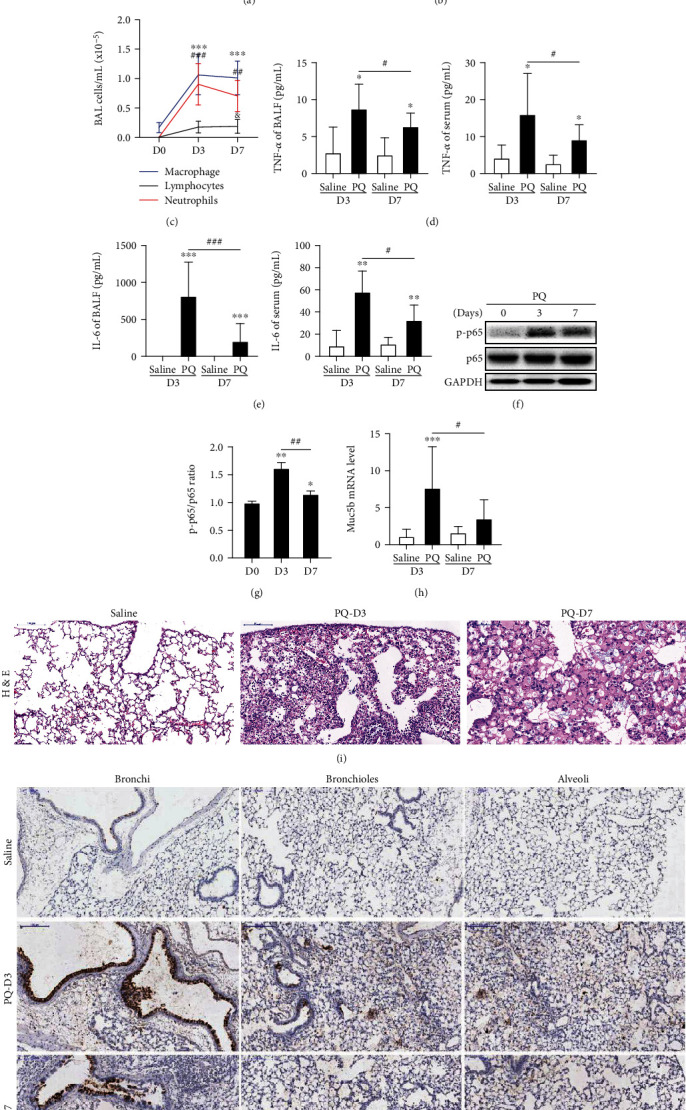
PQ-induced mouse acute lung injury (ALI) activated the NF-*κ*B pathway and induced MUC5B expression. (a, b) Static compliance and resistance of the lung. (c) Quantification of lavaged bronchoalveolar lavage (BAL) cells after PQ instillation. (d, e) Levels of the proinflammatory cytokines TNF-*α* and IL-6 in both BALF and serum. (f, g) phospho-NF-*κ*B p65 from protein level of mice lung tissue. (h) MUC5B mRNA levels of lung tissue were measured by RT-qPCR. (i) Mouse lung sections stained with hematoxylin and eosin (HE). Original magnification: ×200. Scale bar represents 100 *μ*m. (j) IHC staining with Muc5b antibody of injured lung tissues from control or paraquat-treated mice. Original magnification: ×200. Scale bar represents 100 *μ*m. ^&^*p* < 0.05, ^∗^*p* < 0.05, ^∗∗^*p* < 0.01, and ^∗∗∗^*p* < 0.001 compared to the saline control. ^#^*p* < 0.05, ^##^*p* < 0.01, ^###^*p* < 0.001, PQ groups compared between day 3 and day 7. Saline group (*n* = 5), PQ group (*n* = 8) in both day 3 and day 7 experiments.

We first employed a mouse model of PQ-induced lung injury and inflammation and examined the levels of Muc5b expression following PQ treatment (Figure 1). Compared with the saline group, intratracheal aerosolization of PQ induces acute lung injury (ALI) shown by decreased static compliance and elevated resistance of lung (Figures 1(a) and 1(b)), airspace inflammation (Figure 1(c)), and an increase in inflammatory cytokines in BALF and serum (Figures 1(d) and 1(e)). Compared with the saline group, significant neutrophil burst was observed from HE staining at day 3 post PQ treatment (Figure 1(i)). Additionally, PQ also exerted a significant increase of phospho-NF-*κ*B/p65 from protein level compared to the saline group on day 3 (Figures 1(f) and 1(g)). More importantly, measured from both mRNA (Figure 1(h)) and protein levels (Figure 1(j)), Muc5b showed significantly increased levels compared to those in the saline group. On day 7, all the injury and inflammatory parameters, especially the inflammatory cytokines index, were gradually restored but still did not approach that of the saline control mice. The phospho-NF-*κ*B/p65 protein level and the Muc5b level also peaked on day 3 and were partially reversed on day 7, correlating with the inflammatory phenotype.

### 3.2. PQ Activated the NF-*κ*B and MAPK Pathways, Followed by MUC5B Expression and TNF-*α* and IL-6 Production in A549 Cells

**Figure 2:**
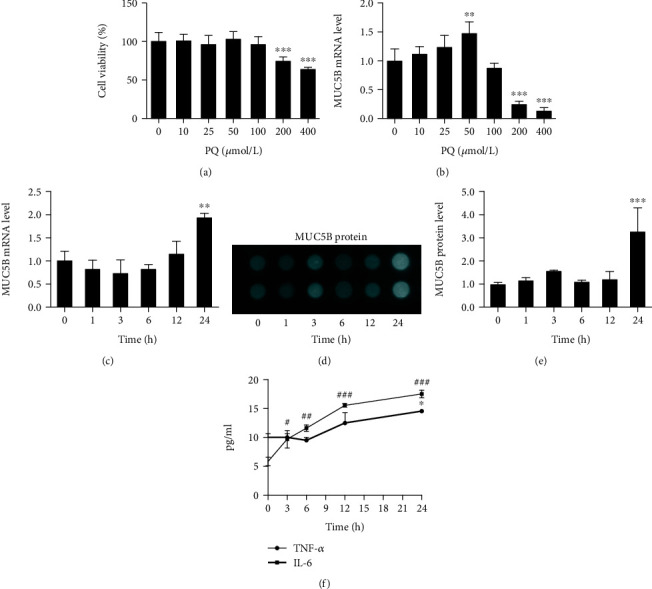
MUC5B expression and TNF-*α* and IL-6 release were increased in PQ-exposed A549 cells. (a) Cells were treated with various concentrations (0, 10, 25, 50, 100, 200, and 400 *μ*M) of PQ for 24 h. Cell viability assay was determined by CCK-8 assays, and the cell viability was not significantly different compared to that of the control groups when the PQ concentration was less than 200 *μ*M. (b) Cells were treated with 50 *μ*M PQ for up to 24 h. *MUC5B* mRNA levels were analyzed by RT-qPCR. *MUC5B* mRNA expression levels gradually increased at 10 and 25 *μ*M PQ and peaked at 50 *μ*M PQ. (c) Cells were incubated with various concentrations (0, 10, 25, 50, 100, 200, and 400 *μ*M) of PQ for 24 h. *MUC5B* mRNA levels were measured by RT-qPCR. (d) MUC5B protein levels were analyzed by dot blot ELISA; samples were duplicate in vertical line. (e) Compared to time 0 h levels in cell lysates, MUC5B protein levels were significantly increased in time 24 h. (f) Cells were treated with 50 *μ*M of PQ for different time points. TNF-*α* and IL-6 levels in the cell culture supernatants were analyzed by ELISA. The expression levels of TNF-*α* and IL-6 were dramatically increased after exposure to PQ for 24 h. The data are shown as the means ± S.D. of three different experiments. ^∗^*p* < 0.05, ^∗∗^*p* < 0.01, ^∗∗∗^*p* < 0.001, compared with no-treatment group (a, b), or compared with the time 0 h (c, e); ^#^*p* < 0.05, ^##^*p* < 0.01, ^###^*p* < 0.001, compared with the time 0 h (f).

**Figure 3:**
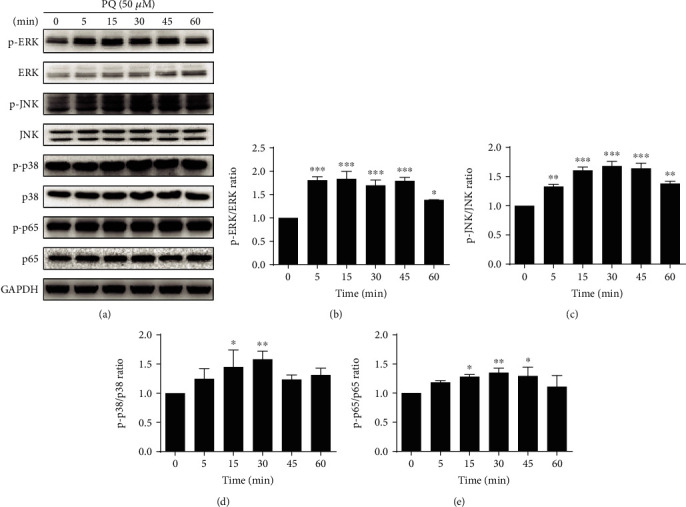
MAPK/NF-*κ*B signaling pathway activation by PQ exposure. (a) Cells were treated with 50 *μ*M PQ for a time-dependent experiment (0, 5, 15, 30, 45, and 60 min). Phosphorylation levels of ERK, JNK, p38, and p65 were detected by Western blot analysis. Quantitative data are provided. The phosphorylated forms of (b) ERK, (c) JNK, (d) p38, and (e) p65 all start increased from 5 min after PQ treatment and significantly at 30 min (p-p65/p65, p-JNK/JNK, p-p38/p38) or 15 min (p-ERK/ERK). The results are expressed as the means ± S.D. of three different experiments. ^∗^*p* < 0.05, ^∗∗^*p* < 0.01, ^∗∗∗^*p* < 0.001, compared with the time 0 h.

We next performed a more detailed kinetic study of PQ-induced MUC5B expression in relation to inflammatory signaling pathways using the human A549 lung epithelial cell line. We first examined the viability of A549 cells after treatment with increasing concentrations (0, 10, 25, 50, 100, 200, and 400 *μ*M) of PQ for 24 h. The results indicated that the cell viability was not significantly different compared to that of the control groups when the PQ concentration was less than 200 *μ*M (Figure 2(a)). Then, the cells were exposed to PQ in dose-dependent and time-dependent manners to examine MUC5B expression. Compared to MUC5B mRNA expression at 0 *μ*M PQ, MUC5B mRNA expression levels gradually increased at 10 and 25 *μ*M PQ and peaked at 50 *μ*M PQ (Figure 2(b)), suggesting that MUC5B expression is uncoupled from PQ-induced cell death. We therefore chose 50 *μ*M PQ for the subsequent experiments. For the time-dependent experiment, cells were exposed to 50 *μ*M PQ for 0, 1, 3, 6, 12, and 24 h. The mRNA expression (Figure 2(c)) and the protein levels of MUC5B (Figures 2(d) and 2(e)) were not significantly upregulated until 24 h. This correlated with the expression levels of TNF-*α* and IL-6 measured by ELISA, which peaked after exposure to 50 *μ*M PQ for 24 h (Figure 2(f)). In sharp contrast, when A549 cells were treated with 50 *μ*M PQ for various time points (0, 5, 15, 30, 45, and 60 min) to evaluate the expression levels of proteins in the MAPK and NF-*κ*B pathways, the phosphorylated forms of ERK, JNK, p38, and p65 all showed rapid increases (Figure 3), starting from 5 min after PQ treatment and reaching statistical significance at 30 min (p-p65/p65, p-JNK/JNK, and p-p38/p38) or 15 min (p-ERK/ERK). Taken together, these results suggest that upregulation of MUC5B by PQ is likely downstream of MAPK and NF-*κ*B pathway activation.

### 3.3. Effect of MAPK-NF-*κ*B Signaling Pathway Inhibition on MUC5B Expression

**Figure 4:**
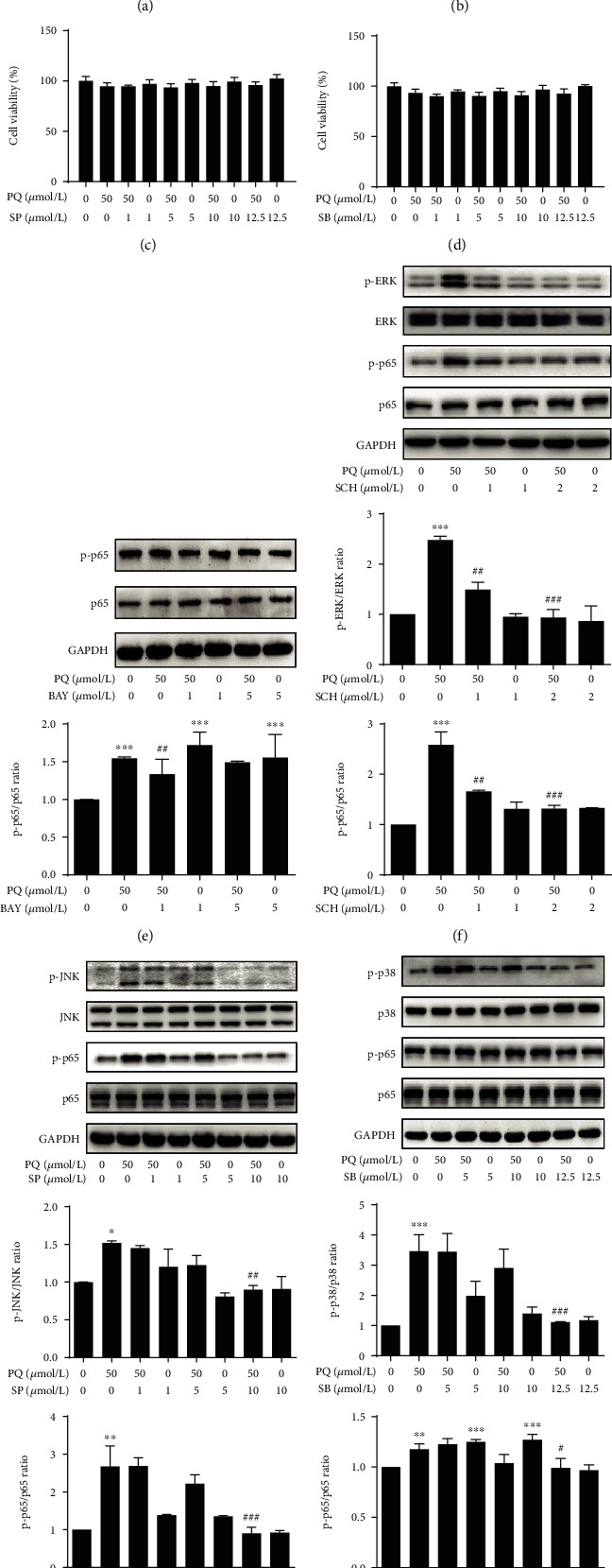
Application of MAPK and NF-*κ*B pathway inhibitors. (a-d) Cells were preincubated with various concentrations of MAPK and NF-*κ*B pathway inhibitors for 2 h and then treated with 50 *μ*M PQ for 30 min. Cell viability assay was determined by CCK-8 assays. (e-h) Phosphorylation levels of p65, ERK, JNK, and p38 were detected by Western blot analysis. The PQ-induced phosphorylation of p65 was signally attenuated in the presence of the p65 inhibitor BAY (5 *μ*M), while the phosphorylation of ERK, JNK, and p38 was observably reduced by the inhibitors SCH (2 *μ*M), SP (10 *μ*M), and SB (12.5 *μ*M). The data are shown as the means ± S.D. of three different experiments. ^∗^*p* < 0.05, ^∗∗^*p* < 0.01, ^∗∗∗^*p* < 0.001, compared with the no-treatment group; ^#^*p* < 0.05, ^##^*p* < 0.01, ^###^*p* < 0.001, compared with the PQ group.

**Figure 5:**
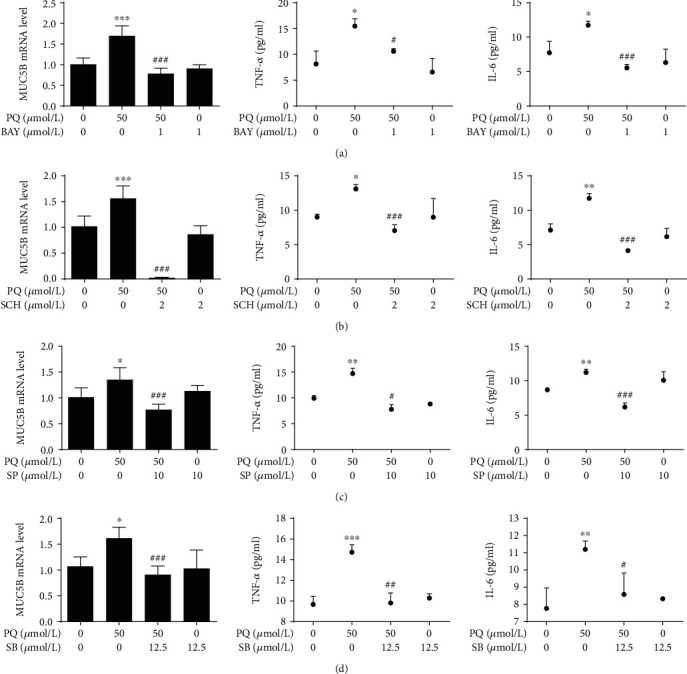
*MUC5B* mRNA expression and TNF-*α* and IL-6 release were decreased via MAPK and NF-*κ*B pathway inhibition in PQ-induced A549 cells. Cells were preincubated with various concentrations of MAPK and NF-*κ*B pathway inhibitors for 2 h and then treated with 50 *μ*M PQ for 30 min. Total RNA was analyzed by qRT-PCR. TNF-*α* and IL-6 levels in the cell culture supernatants were analyzed by ELISA. Inhibiting the MAPK-NF-*κ*B signaling pathway by inhibitors (a) BAY, (b) SCH, (c) SP, and (d) SB all decreased the expression of MUC5B and the extracellular factors TNF-*α* and IL-6 at 24 h after treatment with 50 *μ*M PQ. The data are shown as the means ± S.D. of three different experiments. ^∗^*p* < 0.05, ^∗∗^*p* < 0.01, ^∗∗∗^*p* < 0.001, compared with the no-treatment group; ^#^*p* < 0.05, ^##^*p* < 0.01, ^###^*p* < 0.001, compared with the PQ group.

**Figure 6:**
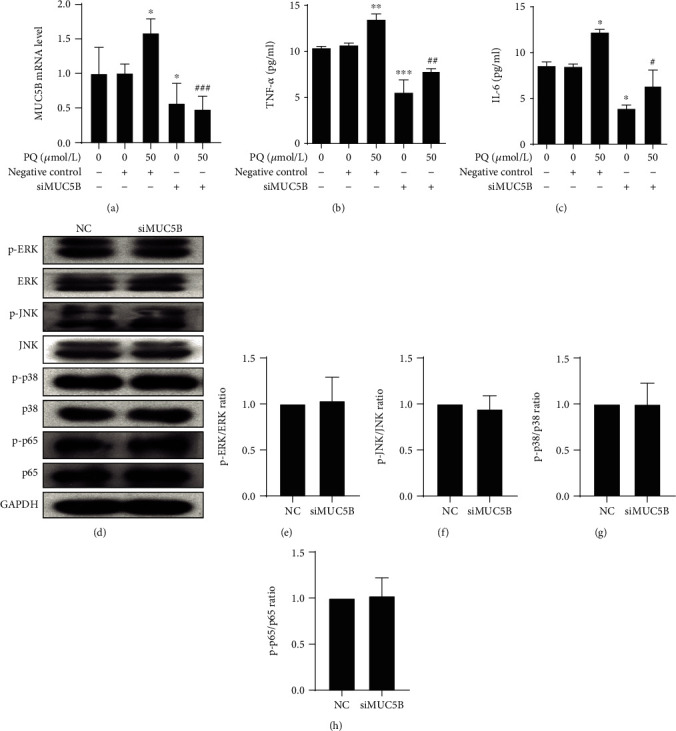
MUC5B regulates the release of TNF-*α* and IL-6. A549 cells were transfected with siRNA against MUC5B for 24 h, and PQ was then added for the cytokine test. Phosphorylation levels of ERK, JNK, p38, and p65 were detected by Western blot analysis (d). Quantitative data are provided. (a) MUC5B mRNA expression was detected by RT-qPCR. MUC5B gene expression levels were significantly lower in the siMUC5B+PQ group than in the negative control+PQ group. (b, c) The release of TNF-*α* and IL-6 into the cell culture medium was detected by ELISA and was also significantly downregulated in the negative control+PQ group. The phosphorylated forms of (e) ERK, (f) JNK, (g) p38, and (h) p65 all kept having the same trend after treatment with siRNA of MUC5B compared with negative control (NC). The results are expressed as the means ± S.D. of three different experiments. ^∗^*p* < 0.05, ^∗∗^*p* < 0.01, ^∗∗∗^*p* < 0.001, compared with the no-treatment group; ^#^*p* < 0.05, ^##^*p* < 0.01, ^###^*p* < 0.001, compared with the negative control+PQ group.

Our kinetic studies predict that the expression of MUC5B may be regulated by the MAPK and NF-*κ*B pathways. To test this hypothesis, we first selected the appropriate concentrations of inhibitors of the MAPK-NF-*κ*B signaling pathway that did not significantly affect A549 cell viability alone or in combination with PQ (Figures 4(a)-4(d)). The PQ-induced phosphorylation of p65 was signally attenuated in the presence of the p65 inhibitor BAY (5 *μ*M) (Figure 4(e)), while the phosphorylation of ERK, JNK, and p38, was markedly reduced by the inhibitors SCH (2 *μ*M), SP (10 *μ*M), and SB (12.5 *μ*M), respectively (Figures 4(f)-4(h)). We next examined the expression of MUC5B under these conditions and found that inhibiting the MAPK-NF-*κ*B signaling pathway significantly decreased the expression of MUC5B as well as TNF-*α* and IL-6 at 24 h after treatment with 50 *μ*M PQ (Figure 5). Furthermore, the phosphorylated forms of ERK, JNK, p38, and p65 were all kept having the same trend after treatment with siRNA of MUC5B compared with negative control (Figures 6(d)-6(h)). The results indicated a model in which PQ treatment activates the MAPK pathway, which in turn initiates a signaling cascade leading to the increased expression of MUC5B and production of inflammatory cytokines.

### 3.4. siRNA Depletion of MUC5B on PQ-Induced TNF-*α* and IL-6 Release

In order to determine causal relationship between MUC5B and PQ-induced inflammation, A549 cells were transfected with negative control or siRNA against MUC5B (siMUC5B), followed by treatment with 50 *μ*M PQ for 24 h. We confirmed the potency of siRNA used by showing that MUC5B gene expression levels were significantly lower in the siMUC5B+PQ group than in the negative control (NC)+PQ group (Figure 6(a)). Importantly, ELISA results demonstrated that the levels of inflammatory cytokines TNF-*α* and IL-6 after PQ treatment were also significantly downregulated upon depletion of MUC5B (Figures 6(b) and 6(c)). Together, these results indicate that MUC5B is indispensable for PQ-induced cellular inflammation.

### 3.5. NAC Attenuates PQ-Induced ERK/JNK MAPK-NF-*κ*B-MUC5B Signaling Pathway and Cell Inflammation

**Figure 7:**
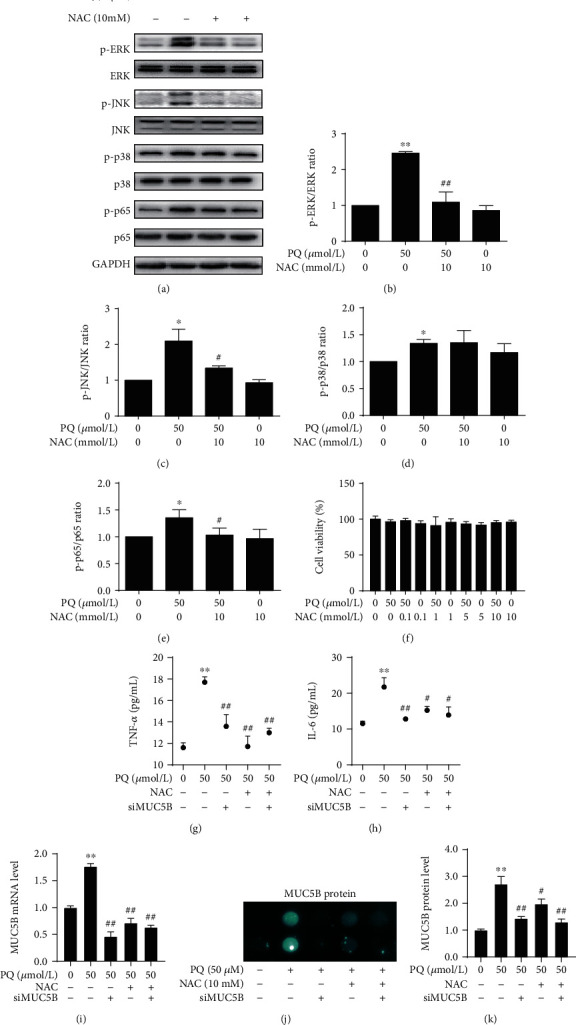
NAC involved in the PQ-induced ERK/JNK MAPK-NF-*κ*B-MUC5B signaling pathway and attenuated MUC5B expression and cell inflammation. Cells were preincubated with 10 mM NAC for 2 h and then treated with 50 *μ*M PQ for 30 min. (a) Phosphorylation levels of ERK, JNK, p38, and p65 were detected by Western blot analysis. Quantitative data are provided. (b-d) The addition of NAC efficiently suppressed the proteins ERK, JNK, and p65 phosphorylation level. (e) However, there was no significantly changes on p38 phosphorylation. (f) Cells were preincubated with various concentrations of NAC for 2 h and then treated with 50 *μ*M PQ for 24 h. Cell viability assay was checked by CCK-8 assays. The cell viability was not changed neither by the PQ nor by the NAC treatment. (g, h) TNF-*α* and IL-6 levels in the cell culture supernatants were analyzed by ELISA. The expression levels of TNF-*α* and IL-6 were dramatically decreased after NAC. (i) MUC5B mRNA was analyzed by qRT-PCR technique and was suppressed by NAC treatment. (j) MUC5B protein levels were analyzed by dot blot ELISA; samples were duplicate in vertical line. (k) Compared to time PQ group, MUC5B protein levels were significantly decreased in the NAC treatment group. When compared with the siRNA and NAC groups, there was no significant difference from the suppression inflammatory cytokine level or MUC5B level between the single NAC treatment group and with the siRNA group. The data are shown as the means ± S.D. of three different experiments. ∗∗*p* < 0.01, ∗∗∗*p* < 0.001, compared with the no-treatment group; ^#^*p* < 0.05, ^###^*p* < 0.001, compared with the PQ group. The data are shown as the means ± S.D. of three different experiments. ^∗^*p* < 0.05, ^∗∗^*p* < 0.01, compared with the no-treatment group; ^#^*p* < 0.05, ^##^*p* < 0.01, compared with the PQ group.

NAC is a commonly used clinical medication to prevent acute inflammatory process induced by PQ. Accordingly, we examined the protein expression levels of phosphorylated ERK, JNK, p38, and p65 (Figure 7(a)) and found that compared to PQ treatment alone, the addition of NAC suppressed MAPK-NF-*κ*B pathway key proteins ERK, JNK, and p65 phosphorylation level (Figures 7(b), 7(c), and 7(e)). However, there was no significant changes on p38 phosphorylation (Figure 7(d)). We confirmed that the concentration of NAC selected did not significantly compromise A549 cell viability (Figure 7(f)). The production of cytokines TNF-*α* and IL-6 (Figures 7(g) and 7(h)) and the mRNA expression levels (Figure 7(i)) and the protein levels of MUC5B were next determined (Figures 7(j) and 7(k)). As expected, NAC could effectively suppress PQ-induced upregulation of MUC5B at both mRNA and protein levels. Furthermore, treatment of NAC could not further reduce the levels of proinflammatory cytokines in cells depleted of MUC5B (Figures 7(g)-7(k)). These results indicated that NAC attenuated PQ-induced A549 cell inflammation by downregulating the ERK/JNK MAPK-NF-*κ*B-MUC5B signaling pathway, and that siRNA-mediated knockdown is sufficient to phenocopy the treatment effect of NAC.

## 4. Discussion

In this study, we employed PQ-treated A549 lung cells to establish an acute inflammation model. Our findings demonstrated that the MAPK-NF-*κ*B-MUC5B pathway is involved in the mechanism of PQ-induced cell inflammation. Importantly, we provide evidence that NAC could effectively reduce cell inflammation by downregulating the MAPK-NF-*κ*B-MUC5B pathway.

PQ ingestion leads to respiratory failure and death [25]. PQ accumulates mainly in the lung, resulting in acute lung injury and pulmonary fibrosis [26, 27]. In the lung, particularly high levels of PQ accumulate in human bronchial epithelial cells, as well as in alveolar type I and type II epithelial cells [28, 29]. MUC5B, secreted by goblet cells in the human airway and by Clara cells in mice, plays an important role in airway defense [9]. Thus, there might be an interaction between MUC5B secretion and PQ uptake in the lungs. Muc5b overexpression causes mucociliary dysfunction and enhances lung fibrosis in mice [22]. These findings are consistent with the essential role of MUC5B in PQ-induced lung injury established here. Our findings also agree with clinical observations that plasma concentration of MUC5B is correlated with outcomes in Paraquat-exposed patients. Moreover, MUC5B was reported to modulate the expression of inflammatory factors [30]. Similar results were also obtained in our study, where MUC5B depletion decreased TNF-*α* and IL-6 release in PQ-induced A549 cells, indicating that PQ-induced cell inflammation was tightly linked connected with increased MUC5B levels. The novel findings that MUC5B participants in the PQ-induced cell inflammation process and is mediated by the MAPK-NF-*κ*B pathway activation warrant further investigation in vivo using Muc5b genetic knockout mouse models.

NAC is used as a mucolytic agent because it can break the disulfide bond of mucin in sputum to decompose mucin; thus, it reduces the sputum viscosity and liquefies the sputum to make coughing easy [31, 32]. A recent study has shown that NAC could reduce the disulfide bond in MUC5B [22]. In addition, other studies have revealed that NAC could reverse the release of inflammatory cytokines [33, 34]. In this study, we confirmed that in a PQ-induced cell inflammation model, NAC significantly reduced the release of TNF-*α* and IL-6. Furthermore, NAC could also downregulate the mRNA expression of *MUC5B*, uncovering another layer of regulation besides suppressing the MUC5B protein level. Depletion of MUC5B was sufficient to phenocopy NAC treatment, suggesting that it is an important mediator of NAC's therapeutic effect.

Inflammatory responses are regulated by MAPK and NF-*κ*B pathway activation [35–37]. It has been reported that PQ can activate the MAPK and NF-*κ*B pathways [38, 39]. Furthermore, suppressing the ERK MAPK pathway could significantly block resistin-induced *MUC5B* mRNA expression [40]. Here, we found that inhibition of ERK/JNK MAPK and NF-*κ*B pathways resulted in a significant decrease in MUC5B. These findings imply MUC5B expression is controlled by the ERK/JNK MAPK-NF-*κ*B pathway. Interestingly, MUC5B could be blocked better with an ERK MAPK pathway inhibitor than with a JNK MAPK pathway inhibitor. Future studies are required to fully elucidate the direct mechanisms for MUC5B activation. .

In conclusion, we utilized in vitro and in vivo models of PQ-induced lung inflammation to investigate the possible role of MUC5B. Expression of MUC5B not only correlated with] but also critically contributed to PQ-induced inflammatory responses. Furthermore, MUC5B likely represents a key target of NAC for its anti-inflammatory effects. These findings support further preclinical and clinical development of MUC5B as a biomarker and therapeutic target for PQ poisoning.

## Figures and Tables

**Table 1:** Primers' information for the real-time quantitative PCR.

Species	Gene	Forward (5′-3′)	Reverse (5′-3′)
Mouse	Muc5b	AGGATGGGCAGCAGAAACTG	TCTGACTGTCTCCGGTGAGTTC
Human	MUC5B	AGTTTCCGTCCTTGTCGTAGC	CTGCCCCTTGTTCTGTGACTT
Mouse	IL-6	TAGTCCTTCCTACCCCAATTTCC	TTGGTCCTTAGCCACTCCTTC
Human	IL-6	GCCAGAGCTGTGCAGATGAG	TCAGCAGGCTGGCATTTG
Mouse	TNF-*α*	CACCACGCTCTTCTGTCT	GGCTACAGGCTTGTCACTC
Human	TNF-*α*	CTCAGCAAGGACAGCAGAGG	ATGTGGCGTCTGAGGGTTGTT
Mouse	GAPDH	AAGAAGGTGGTGAAGCAGG	GAAGGTGGAAGAGTGGGAGT
Human	GAPDH	GGACCTGACCTGCCGTCTAG	GTAGCCCAGGATGCCCTTGA

## Data Availability

The data used to support the findings of this study are available from the corresponding author upon request.

## Supplementary Materials

Supplementary Materials Supplementary Table 1: antibodies applied.
