# Supplementary material for: The MUC5B Mucin Is Involved in Paraquat-Induced Lung Inflammation
Source: Oxid Med Cell Longev. 2020 Jul 16;2020:7028947. doi: 10.1155/2020/7028947 (PMC7381986; doi:10.1155/2020/7028947)
Supplement: Supplementary Materials — Supplementary Table 1: antibodies applied. [file 7028947.f1.docx]

**Supplementary Table 1** **Antibodies Applied**

| Primary antibody | Host species and catalog number | Company |
| --- | --- | --- |
| p-JNK | Rabbit #9251 | Cell Signaling Technology |
| JNK | Rabbit #9252 |  |
| p-ERK | Rabbit mAb #4370 |  |
| ERK | Rabbit #9102 |  |
| p-p38 | Rabbit #9211 |  |
| p38 | Rabbit #9212 |  |
| p-p65 | Rabbit mAb #3033 |  |
| p65 | Rabbit mAb #8242 |  |
| GAPDH | Mouse T0004 | Affinity |
